# Knowledge, Attitudes, and Barriers Toward Research Among Medical Students of Karachi

**DOI:** 10.7759/cureus.5599

**Published:** 2019-09-09

**Authors:** Muhammad Bilal, Abdul Haseeb, Anum Mari, Sana Ahmed, Muhammad Ahad Sher Khan, Muhammad Saad

**Affiliations:** 1 Internal Medicine, Dr Ruth KM Pfau Civil Hospital, Karachi, PAK; 2 Internal Medicine, The Wright Center for Graduate Medical Education, Scranton, USA; 3 Internal Medicine, Ziauddin Medical University, Karachi, PAK; 4 Internal Medicine, Dr Ruth K M Pfau Civil Hospital, Karachi, PAK; 5 Internal Medicine, University Hospital Kerry, Tralee, IRL

**Keywords:** medical students, knowledge, attitude, barriers, research

## Abstract

Introduction

Our study was meant to assess the knowledge, attitude, and barriers towards research in medical students of Pakistan. By assessing the factors, we aim to increase the role of medical students in research, which will eventually help developing countries like Pakistan to achieve self-reliance in health care.

Methods

Undergraduate and postgraduate students of medicine, dentistry, and pharmacy schools of Dow University of Health Sciences, Karachi, were enrolled from February-March 2018 in a cross-sectional, descriptive study using questionnaires to provide details of the parameters of attitude to the knowledge of and barriers towards research for each individual. All data were coded for each of the parameters. Data analyses were performed by one-way analysis of variance (ANOVA)/Tukey and Student’s t-test, Pearson’s correlation, and Chi-squared tests.

Results

A total of 850 questionnaires were received. The overall mean scores of students on attitude, knowledge, and barriers were 69.27 ± 13.44, 70.39 ± 15.67, and 72.46 ± 13.46, respectively; 81.8% of students’ scores fell above the middle of the maximum score for knowledge, but 84.5% of attitude scores came in at below the middle of the maximum score. Undergraduate students had a more positive attitude to research than postgraduate students (69.20 ± 11.10 vs 64.23 ± 10.98; p = 0.002). Male students had a better attitude than females (72.97 ± 20.54 vs 67.09 ± 21.56; p = 0.010). Barriers highlighted by students most significantly included a lack of funding support and preference for instruction over research.

Conclusion

Students showed good knowledge of research, but their attitude was not up to the mark. The barriers highlighted suggest a need for a change in the strategies for research. Attention should be paid to inculcate research as part of the student curriculum and to make available incentives, information, and mentors to solve the problems most students face in the field of research.

## Introduction

In contemporary times, where advancements in the field of medicine are taking place at a never-before pace, it has become an essential requirement to stay updated with progressing medical techniques. Therefore, health research has become an important component of medical education. Medical research not only ingrains the art of critical thinking and reasoning skills in a health professional but also provides new findings having the potential to influence health care. Thus, motivating medical students towards research in the early years of their medical career can help developing countries like Pakistan to achieve self-reliance in health care and aid in the process of development [1]. Research is also considered to be one of the best indicators of the scientific progress of a country [2]. It was found that the increased participation of undergraduate medical students has a positive impact on their career choice, oral/written communication skills, and analytical thinking [3]. Further, these projects help in producing physicians better accustomed to applying new knowledge to their profession [4]. Insufficient attention to research by the government and well-educated population of a community may contribute to the scientific and knowledge lag within the national community and world as a whole and, therefore, concern over conducting scientific and accurate research has grown over the past years in most countries [5-6]. In Pakistan, the number of physician-scientists has declined over the past two decades and there is a dire need for more clinical as well as basic health science investigators given the ever-rising importance of medical research.

One of the most important predictors of research attitude is the researcher’s beliefs about the particular subject of research, as they influence his motivation level to conduct research in the first place. The majority of researcher’s beliefs are found to be positive in countries like Ireland, Croatia, New Zealand, and Pakistan [7-9]. Barriers to research among medical students were found to include: inadequate knowledge of study design, time limitations, restricted funding support, lack of research training, lack of mentors, lack of research self-efficacy, lack of interest, and limited access to data sources [6]. In Pakistan, knowledge of research is lower during the initial years of medical school and students undergoing lecture-based learning generally show less interest in health research than those undergoing problem-based learning [9]. Furthermore, factors like curriculum overload, Internet inexperience, an uncooperative community, difficulty in finding a mentor, difficulty in selecting a topic, and lack of previous exposure lead to a lack of interest in research among medical students of Pakistan [10].

Given the emerging role of research in health care, it is imperative to conduct studies that signify the current status toward conducting research. Therefore, our study is meant to assess the knowledge, attitude, and barrier towards research in medical students of Pakistan. By assessing these factors, we can help make research more appealing to medical students to increase the number of skilled researchers in the future.

## Materials and methods

In this study, we surveyed the opinions of fourth and final year medical students towards research to find out the predictors of attitude and barriers to research among medical students.

Our study sample consisted of fourth and final year medical students of the academic year 2018 of Dow Medical College. The duration of the study was two months from February-March 2018. The total sample size was 850.

We distributed a pre-validated, three-page, self-reporting questionnaire to medical, dental, and pharmacy students [6]. The questionnaire consisted of three main sections. The first section included demographics, i.e. gender, marital status, year of birth, year of entry, undergraduate or postgraduate student, and the field of education, e.g. medical, dental, or pharmacy. The second section included questions that were used to assess attitude. The 5-point Likert rating scale was used to score the answers. The questions were based on the perceived importance of scientific research in the students’ mind in aspects of their education, life, and medical career and their reasons of being interested in research, kind of research they would prefer, and, finally, their future plans to take part in research. The third section assessed knowledge of research through eight questions varying from the basic knowledge of kinds of scales, knowledge of the writing styles of references, scientific writing, to database resources. The last section focused on the barriers toward research that were assessed through 32 questions. The questions focused on the factors limiting the student’s role in research, whether it was lack of research ideas, problems in performing research (i.e. lack of access to equipment and research materials or the lack of suitable research space), lack of time, etc.

The content of the questionnaire was adapted from a previous similar study, with efforts made to make the questionnaires appropriate to our local university [6]. Students have explained the objectives of the study and reassured confidentiality. All the data collected from medical students were anonymous except for their year of study. Informed written consent was taken. The questionnaire, along with the protocol of the study, was approved by the Institutional Ethical Review Committee of Dow University of Health Sciences Karachi, Pakistan. No conflict of interests was encountered in the entire study period. A pilot study was conducted with 30 medical students, to rule out any ambiguity in the questionnaire, and the pilot study data were not included in the final analysis. Minor changes were made in the questionnaire after the pilot study had been conducted.

All of the responses were coded for each of the parameters. The students’ answers were compared to each other to find any impact of age, sex, marital status or level of education on their responses. Data analyses were carried out using SPSS (IBM Corp, Armonk, NY, US). One-way analysis of variance (ANOVA)/Tukey was used to compare the mean scores and Student’s t-tests were used for ages in different levels and fields of education. Pearson’s correlation coefficient was used to assess the relationship between age and scores. The chi-square test was used to make comparisons between groups of different sex and marital status. In this study, p < 0.05 was considered statistically significant.

## Results

Completed forms from 850 undergraduate (mean age 22.55 ± 3.50) and 250 post-graduate students (mean age 30.15 ±4.56) were taken into account. In total, 67% were women and 33% were men. Table 1 shows the demographic data for participant students. The overall mean scores of students on attitude, knowledge, and barriers were 69.27 ± 13.44, 70.39 ± 15.67, and 72.46 ± 13.46, respectively.

**Table 1:** Demographic data for students participating in the study

		Total Participants (N=850)	Undergraduate Students (N=600)	Postgraduate Students ( N=250)
Gender	Male	280	150 (25.0 %)	130 (52.0%)
	Female	570	450 (75.0 %)	120 (48.0%)
Marital Status	Single	582	432 (72.0%)	150 (60.0%)
	Couple	268	168 (28.0%)	100 (40.09%)
Field of Education	Medical	450	300 (66.6%)	150 (33.3%)
	Dental	260	200 (76.6 %)	60 (23.1%)
	Pharmacy	140	100 (71.4%)	40 (28.6%)

Table 2 shows comparative means between the sex and marital status groups as well as field and level of education groups. Male students had a better attitude than females (73.10 ± 11.1 vs 69.65 ± 13.06; p=0.015), and single students had a better attitude than their married peers (70. 10± 11.31 vs 64.22 ± 12.21; p=0.03). The mean attitude score of undergraduate students was significantly greater than that of postgraduate students (69.20 ± 11.10 vs 64.23 ± 10.89; p = 0.002). However, there was no significant difference between the education levels of students in terms of their knowledge and barrier scores (p =0.434; p = 0.546). The mean for knowledge in dental students was significantly lower than that of pharmacy and medicine students (p = 0.002). There was no significant difference between the educational field of students and their attitudes and barriers (p=0.206, p=0.345).

**Table 2:** Comparison of mean (± SD) of research subjects for demographic characteristics In the field of education, different letters in each column show significant differences between the fields (Tukey HSD (honestly significant difference) test). p<0.05 is significant*.

Variable		Attitude	Knowledge	Barrier
Sex	Male	73.10±11.01	65.31±11.23	72.32±14.65
	Female	69.65±13.06	71.98±10.67	73.10±11.21
P-value		0.015*	0.672	0.343
Marital Status	Single	70.10±11.31	71.22±20.36	76.22±16.22
	Couple	64.22±12.21	69.32±13.30	71.23±14.23
P-value		0.030*	0.675	0.333
Field Of Education	Medical	72.21±12.20	72.23±12.21	74.20±21.20
	Dental	65.23±11.09	63.32±33.20	72.08±33.32
	Pharmacy	67.21±11.90	77.20±11.09	73.32±12.28
P-value		0.206	0.002*	0.345
Level of education	Undergraduate	69.20±11.10	73.20±13.21	72.20±22.20
	Post Graduate	64.23±10.98	72.32±10.98	69.21±23
P-value		0.002*	0.434	0.546

Table 3 shows comparative means and standard deviations between the three different fields, i.e. medical, dental, and pharmacy and level of education, in terms of undergraduate or postgraduate, on research subjects. The levels for attitude, knowledge, and barriers to health research, assessed by the percentage of students falling in the quartiles of the possible score for each field as indicated in Table 4. Data showed that most of the student (84.5%) had an attitude score to research that was lower than half of the maximum score while 81.8% fell above half of the maximum score on the knowledge parameter and showed ample knowledge.

**Table 3:** Comparison of mean (±SD) of research subjects for educational characteristics UGS: undergraduate students, PGS: postgraduate students p < 0.05 is significant.

Field	Educational Level	Attitude	p-value	Knowledge	p-value	Barrier	p-value
Medical	UGS	72.21±11.11	0.233	4.20±1.1	0.323	75.09±8.11	0.232
	PGS	66.67±13.22		4.30±1.33		72.23±7.99	
Dental	UGS	67.21±23.32	0.015*	7.33±2.33	0.987	74.20±12.11	0.545
	PGS	63.22±12.23		5.23±3.20		70.23±13.11	
Pharmacy	UGS	68.21±13.31	0.060	5.05±2.30	0.656	73.43±12.11	0.898
	PGS	62.23±14.48		5.10±2.11		75.13±61	

**Table 4:** Distribution of attitude, knowledge, and barrier scores of participants regarding the percentages of total attainable score

Percent of total score	Attitude	Knowledge	Barriers
<25%	85 (10.0%)	60 (7.1%)	187(22.0%)
26%-50%	634 (74.5%)	94(11.1)	612(72.0%)
51%-75%	51 (6.0%)	425(50.0%)	34(4.0%)
>75%	80 (9.4%)	271(31.8%)	17(2.0%)

Table 5 shows the highest percentage of respondents in several fields of barriers toward research. Inadequate financial support was found to be a major barrier (87.9%). This was followed by a preference for academic instruction over research (79.5%), lack of skills and knowledge (77.5%), and lack of incentive (75.3%).

**Table 5:** Percentage of main barriers toward research by medical students

Lack of incentive	75.3%
Lack of mentorship support	62.5%
Lack of researcher acknowledgment	66.0%
Researcher economic problems	71.5 %
Lack of skill and knowledge	77.5%
Limited time	72.5%
Preference of instruction over research	79.5%
Lack of funding support	87.5%

## Discussion

Recent advances in medicine and health care have made research an integral part of modern medicine. However, in many developing countries like Brazil, research is not yet a compulsory component of medical education although it is well-known to have a positive impact on the quality of medical education [11]. Apart from a few studies aimed at investigating the involvement of medical students in research and the barriers they face, research training in developing countries, especially Pakistan, have not yet been adequately addressed. This study is an attempt to better understand the current understanding of research and the barriers that students, both undergraduate and postgraduate, face in implementing research knowledge to conduct research during their medical education.

With regards to attitude towards research, the results of our study showed that most of the students had an attitude to research that was lower than half of the possible attainable score, which is inconsistent with what other studies have found, e.g. Vodopivec et al. [7] and Khan et al. [1]. This may be because of the difference between the location of the study and the questions asked in our study to assess attitude. While the attitude score was found to be relatively lower among our study participants; most fell above half of the possible attainable scores on the knowledge parameter and showed adequate knowledge in our study. Our study showed that male students had a more positive attitude than their female peers, but knowledge of research was not different between genders. Similar results were obtained by studies in the US and other studies conducted in Pakistan [9,12]. In contrast to our study, Amin et al. [13] and Memarpour et al. [6] concluded in their study that females had better knowledge than males while attitudes were indifferent between the two genders. The differences may be attributable to differences in sample size, in population, or to the increased ratio of acceptance of female students in Pakistan’s medical universities when compared to their male counterparts.

According to a study in the UK, uptake of research opportunities by undergraduates is disappointing [14], however, the results of our study showed the mean attitude score of undergraduate students was significantly greater (compared to postgraduate students) and increasing age, level of education, and marriage were clearly seen to have an adverse effect on attitude to and knowledge of research. The findings of Askew et al. [15], Khan et al. [1], and Memarpour et al. [6] were similar to ours, reporting that physicians of a younger age were more keen about research. This may be because the schedule and pressure involved in further studies drastically decrease the motivation and time available to postgraduate students to avail research, along with marital responsibilities [12] and the perception that research has no more than a minimal role in their profession [9]. However, Vukaklija et al. noted the opposite, as they found the attitude toward research enhanced among undergraduate students with each year of education [2]. This difference may be because the level of education of students evaluated was different in their study as compared to ours or because of variations according to the country of study.

Our study results found the mean for knowledge in dental students to be significantly lower than that of pharmacy and medicine students. This differs from the findings of Memarpour et al. [6], who found medical students to have less knowledge than dental or pharmacy students. This particular difference could be due to a difference between the two studies in the ratio of students sampled from each school.

Previous studies have documented a decline in the number of physicians-scientists in medical practice [16]. Quite a few studies have already proven a lack of interest as a general trend among medical students, leading to a lack of commitment to publishing an article [1,17]. Even among postgraduates, factors associated with an observed decline in the physician-scientists range from a lack of incentives to inadequate prior exposure to medical research during undergraduate years [18-19]. As such, this decline in interest can be attributable to a number of factors, including a paucity of institutional incentive, time restraints, a lack of practical application of research methodology, failure to understand the important role that research plays in our community, and the availability of mentors to overlook such projects, difficulty in finding a mentor [20], however, our study results showed that only 62% of students regard lack of mentorship as a barrier to conducting research and almost 88% regarded inadequate financial support as the most significant barrier to their participation in research activities, followed by a preference for academic instruction over research, lack of skill, and lack of incentive. Similar to our study, a lack of research funding support was also cited as the most important barrier for students in other studies [13,21]. This may be because the limits on funding for research allowed a low incentive for research [9].

An interesting finding of our study is that even though only 18% of students had knowledge below half of the possible attainable score, almost 78% perceived a lack of knowledge and skill as a barrier towards research. This means that even when students have adequate knowledge of research, they perceive it to be little or less than that required to practically take part in or carry out their own research. This barrier can be addressed by creating positive awareness of research among students and empowering them to carry out their research by mentoring them through all the steps of research. Limited time due to workload can be addressed by making a slot specifically allocated for research activity in the student curriculum. For research funds, institutes should try to avail funding support not only from the government but also other resources and researches should be told to look out for grants and awards.

The study was conducted at one institution to serve as a pilot for other studies that should be conducted on a larger scale. Thus, the findings cannot be generalized for the whole population of Pakistani medical students. Despite these limitations, the use of a validated questionnaire allows for a comparison of our findings to other studies done under similar settings and using the same evaluative tool. We encourage further detailed studies to be carried out across health institutes all over the country to address this crucial issue of research. Further, other factors influencing research activity and barriers towards conducting research, such as funding, research infrastructure, increasing the cost of medical education, and adequate research opportunities, need to be evaluated.

## Conclusions

In conclusion, we report that medical students from schools of medicine, dentistry, and pharmacy had adequate knowledge of research, which did not improve with the year of training, although their attitude toward the research process was below average. The results of this study have been able to deduce that medical students require motivation and financial support to get involved in research projects. Attention should be also be directed toward inculcating research as a part of undergraduate student curriculum and to make available incentives, information, and mentors in order to solve the problems most students face in the field of research.
